# Conference Report: NIST-INDUSTRY WORKSHOP ON THERMAL SPRAY COATINGS RESEARCH Gaithersburg, MD July 20, 1992

**DOI:** 10.6028/jres.098.030

**Published:** 1993

**Authors:** S. J. Dapkunas

**Affiliations:** National Institute of Standards and Technology, Gaithersburg, MD 20899-0001

## 1. Introduction

In 1992, a comprehensive survey of current Federal materials research programs and plans for new initiatives, known as the Advanced Materials and Processing Program (AMPP), was developed under the auspices of the Office of Science and Technology Policy. The goal of the AMPP is to improve the manufacture and performance of materials to enhance the nation’s quality of life, security, industrial productivity, and economic growth [1]. One element of the program addresses what have become known as “Functionally Gradient Materials” (FGM). This class of materials is distinguished by properties which vary with material thickness. These gradients in properties such as hardness, thermal conductivity and chemical stability contribute to the improved performance of components. For example, the wear resistance of a soft but tough material can be greatly improved by the application of a harder but compliant surface deposit overlayed with a very hard but brittle material. The concept of gradations of material properties to optimize performance is not new but increased awareness of the opportunities for improved control of properties and the ability to tailor microstructures continuously through a thickness has fostered a view of these materials as a distinctive class whose potential has not been fulfilled. Typically these materials’ compositions vary from metallic to ceramic over a thickness of up to several millimeters. The unique approach now taken to graded materials in FGM research is to target specific properties at the extremes of the material’s thickness, thermal conductivity, and thermal expansion, for example, and to tailor microstructure, porosity or other features to meet those properties. Extensive research to achieve this capability through understanding of processing-microstructure-property relationships has been initiated through material synthesis techniques as varied as thermal spray, chemical vapor deposition, and self propagating high temperature synthesis [2].

Thermal spraying of coatings, due to high material deposition rates and relatively low capital cost, has become a primary industrial method of synthesizing materials with varying composition and microstructure. The North American thermal spray market was over $600 million in 1990 and is projected to reach $2 billion per year by the year 2000, a growth rate of 7–8% per year. The largest portions of this market are in powder consumables and coating services. The growth projected is largely based on the increased use of ceramic coatings for thermal barriers and clearance control on aircraft gas turbines, some of which have close to 5500 parts which are thermal spray coated [3]. Similarly, the market for ceramic powders used in thermal spraying is a significant portion of the advanced ceramic powders market and is expected to experience a growth rate of 4% per year through 1995 [4] with annual consumption of oxides and carbides reaching over 2 million kilograms by the turn of the century [5].

The thermal spray industry is diverse. In addition to the aircraft engine applications noted above, thermal spray deposited coatings are applied to fossil fueled boilers and chemical processing vessels to control corrosion, to automotive bodies and mechanical components for cosmetic and wear reduction purposes, to electrical components for insulation, and research is under way to increase efficiency of reciprocating engines through application of thermal barrier coatings to piston crowns. Thermal spray processes are also used to refurbish worn mechanical components, thereby reducing scrap and replacement costs [6]. The companies which supply materials and services for these applications are likewise diverse in size and capabilities. The 1992 Thermal Spray Buyers Guide lists 38 powder suppliers, 25 equipment suppliers, and 40 contract applicators [7] in addition to the major automotive and aerospace companies which are the large single site users of the technology.

Recognition of the size of this industry, the varied interests and skills of the scientists and engineers involved, and the changes in the field have fostered the growth of technical and trade organizations to serve the field’s technical communication needs. The primary domestic technical society addressing the community is the Thermal Spray Division of ASM International, which was formed in 1987 and now sponsors the publication of the Journal of Thermal Spray Research. Trade and marketing issues are served by the International Thermal Spray Association. Although research results are available in a wide venue of scientific and engineering journals, the most comprehensive summaries of current research and technical developments are found in the proceedings of the 13 International Thermal Spray Conferences (1956–1992) and the 4 National Thermal Spray Conferences (1981–1991). These conferences now attract over 1000 attendees each.

## 2. Thermay Spray Processing

Thermal spray coatings are applied by injecting the material to be deposited into a high velocity hot gas directed to the substrate of interest. The coating feedstock is generally a powder but wires and rods are also widely used and the controlled feeding of this material allows the development of a coating whose composition and microstructure varies with thickness. The high temperature gas is obtained either by the development of a plasma generated by passing an inert gas through a set of high voltage electrodes or by combustion of reactive gases in the torch itself. Plasma spraying and flame or high velocity oxygen fuel (HVOF) are the general descriptions of these processes respectively. Process conditions can vary widely and have significant influence on the microstructure and properties of the deposited material. Table 1, taken from Ref. [8], identifies some of the pertinent features of the various thermal spray processes. The plasma spray process has been adapted for operation in vacuum to increase the density of the deposit and in inert gas filled chambers to prevent oxidation of the material sprayed.

The high temperatures and velocities of the thermal spray processes make measurement and control of process parameters difficult. Therefore, although widely used, production of quality coatings largely depends on the experience and intuition of skilled equipment operators.

Microstructures of deposited coatings are complex, particularly for graded structures. The extreme thermal conditions to which feedstocks are subjected, high cooling or solidification rates, reaction during transit to the workpiece (substrate), and morphological features resulting from high but variable impact velocities combine to make prediction and control of microstructures difficult. Hence, much of the material produced commercially is the result of empirical studies relating gross process parameters to microstructures and to performance in actual application. Typical thermal sprayed coatings exhibit overlapping lamella resulting from successive impacting particles of molten or very plastic material, oxide films surrounding the lamella, irregularly distributed porosity, rough interfaces between layers of different composition, and cracks resulting from shrinkage during cooling. These features make quantitative analysis and specification difficult.

Properties of thermal sprayed coatings are difficult to measure, especially in service. Tensile strength, elastic modulus, thermal conductivity, and fracture toughness are important coating properties but the most sought after property is adhesion to the substrate. Typically this is determined by the tensile adhesion test, ASTM-C633-79. Large variations in adhesion strength measured by C633-79 have been shown to be typical [9]. Other properties related to performance, such as erosion or corrosion resistance, are routinely measured and related to operating conditions in specific applications.

## 3. Workshop Objective and Structure

The objectives of the workshop were to identify (1) the research required to improve processing reproducibility and performance prediction, (2) opportunities for collaboration between NIST and industrial researchers, and (3) mechanisms of effective dissemination of research results to the thermal spray community. This approach is more focused than some earlier studies, Refs. [10] and [11] for example, which include thermal spray in general assessments of coating research needs and do not specifically address industrial processing concerns.

Invited attendees represented a broad spectrum of the thermal spray industry including powder suppliers, equipment manufacturers, and applicators and users of thermal spray coatings. In addition, researchers from academia and federal laboratories with active programs in thermal spray, as well as representatives of the principal organizations through which the thermal spray community communicates were invited.

The workshop was structured to present visitors with an overview of unique NIST analytical and materials characterization techniques which are viewed as providing improved capabilities to understand the role of processing on performance and properties. Members of the Materials Science and Engineering Laboratory and the Chemical Science and Technology Laboratory staffs reviewed chemical and compositional mapping of microstructures, thermal properties measurements, and powder characterization techniques developed and utilized at NIST.

A crucial aspect of the workshop was to solicit the view of industry on their requirements for measurement related research. To accomplish this, industrial and academic representatives described the general requirements for measurement of process parameters, mechanical properties, microstructural analysis, and modeling of the thermal spray process. The specific issues of the automotive industry were addressed by representatives from Ford and General Motors, who emphasized performance prediction.

To facilitate implementation of research results, NIST personnel described the various mechanisms, such as Cooperative Research and Development Agreements and the Advanced Technology Program, through which collaborative research can be conducted. Similarly, the past chairman of the ASM International Thermal Spray Division described that organization’s structure and means of coordinating dissemination of information.

Following these general presentations, working groups convened to determine specific research topics which are of importance to the thermal spray industry. These groups addressed process measurement and control with an emphasis on powder characterization, coating evaluation, performance evaluation, and process modeling.

## 4. Research Issues

The following research issues and needs were determined by consensus through the working group discussions.

### 4.1 Processing Measurement and Control

Powders are the predominant form of thermal spray feedstock and are increasingly recognized as having a strong influence on the microstructure and performance of coatings. Therefore, consideration of powder characteristics as a process variable is warranted. Powders for thermal spray deposition are synthesized by several techniques and are generally specified by bulk composition and particle size. Metallic powders are usually formed by atomization from an alloy melt while oxide, carbide and other ceramic compositions are formed by crushing and grinding larger material to the desired size or by spray drying fine powder with an organic binder to obtain the size required. The type of ceramic processing used influences properties such as powder shape, phase content, friability, and flowability.

The powder characterization working group aimed to identify those powders which were of greatest interest to industry, to determine which powder properties were most critical to spray process control, and types of standardized testing which are required or need improved technique.

Powders can be divided into those that are intended for coating use at elevated temperatures and those that are exposed to ambient temperatures. Among the former are the zirconia containing thermal barrier and M-CrAlY (Nickel, Cobalt, and Iron as the primary constituent(s) with Chromium, Aluminum and Yttrium alloying additions) coatings applied to gas turbine components and among the latter are tungsten carbide and aluminum oxide utilized for wear protection. For high temperature applications, 7–8% yttria stabilized zirconia was found to be of greatest interest. For this powder, in particular, synthesis technique has a pronounced effect on powder shape, porosity and other features which affect both spraying and deposit formation. These synthesis related features were felt to be the cause of variations in measurements required for powder specification. Specific working group recommendations for research to resolve these issues are as follows:

- Calibration and cross correlation of powder size measurements by powder producers and users should be conducted using well characterized reference lots of material as has been done in the fine ceramics industry. This would be most effectively conducted through the distribution of captive cells of material. This research would have immediate benefits to the thermal spray industry.
- A standard should be written for the size analysis of powders in the 10 μm size range including sample preparation technique. Major interest focused on the analysis of gas atomized and spray dried powder which should be the primary materials studied. Size analysis of non-spherical powders is not currently conducted in spite of the fact that significant amounts of this material are used. Optical size measurement methods are desired, particularly by users.
- Techniques for measurement of specific surface area, phase composition, and chemical composition require development for thermal spray powders. In particular, analysis techniques for impurities such as silica, alumina, sodium, hafnium, uranium, and thorium in zirconia are needed. Apparent density measurement techniques for spray dried powders require development to allow improved process control.
- The ultimate test of a powder is its behavior in the thermal spray process and the working group opined that development of a standardized spray test which would determine deposition efficiency is warranted. It was the group consensus that an impartial institution such as NIST would be extended cooperation from the thermal spray community to develop such testing procedures.
- Sensors to measure thermal spray process conditions were clearly identified as requiring development. Conditions which require measurement include particle temperature, particle velocity, in-process coating density, and deposit thickness. It was felt that an emphasis should be placed on high velocity spraying techniques.

Significantly, powder producers expressed willingness to contribute to the development of the research recommended through contribution of materials, analysis procedures, and the conduct of analyses as part of a collaborative research effort.

### 4.2 Coating Evaluation

Measurement of coating properties and analysis of coating microstructure and microchemistry are critical to both the evaluation of thermal spray processes as well as the prediction of performance. Working group members identified this broad range of immediately useful research which would enhance productivity and effective application:

- The inhomogeneous microstructure typically produced is difficult to analyze due to the presence of constituents as diverse as gross porosity, interlamellar phases, metallic glasses, and oxides. The situation is made more complex by the presence of metastable phases resulting from rapid solidification of powders upon impact with the substrate. Development of reproducible methods to provide quantitative analysis of both gross and subtle features by optical and electron microscopy are needed. It was suggested that a microstructural atlas of coatings would be of use to industry.
- Development of techniques of x-ray diffraction which can routinely be utilized to determine phase content and composition presents challenges which if overcome could provide improved understanding of the role of processing conditions. Similarly, techniques to quantitatively assess phase fraction, residual stress, grain size, and solute levels requires development. The identification of metastable phases which have weak x-ray diffraction patterns is confounded by accompanying fine grain sizes, internal stresses, solute gradients, and texturing effects. This analysis is not performed although the presence of these features can have a significant effect on performance. Characterization of microcracking, which can have significant effects on strength, fracture toughness, thermal conductivity, and corrosion protection, is not easily or well conducted. Development of methods to analyze microcracking can also provide insight into the mechanisms of coating failure.
- Measurement techniques to determine the mechanical properties of thermal sprayed coatings are not well defined although research addressing this topic has been conducted [12–13]. Adhesion to the substrate, cohesion within a coating, and properties of the coating material, particularly when graded, are important to coating design and understanding of the role of processing parameters. Typically adhesion is measured by use of the tensile adhesion test (TAT, ASTM-C633-79) originally developed for evaluation of zinc coatings on steel. This test, which consists of pulling the coating from the substrate by means of a tab epoxied to the coating, is limited to the strength of the epoxy. It is not conducted above 200 °C and provides only rough quality control guidance in contrast to more elegant techniques applied to homogeneous thin films [14]. A test methodology which can be readily conducted by applicators and provides an understanding of mode of failure, failure initiation site, strain to failure, and other pertinent data for the coating-substrate system is desired. Data on the properties of coating materials is either gathered from handbook values of bulk material of similar composition or measured on coatings removed from a substrate. These data are either not representative of the coating or neglect the role of interfacial constraint at the substrate.
- Thermal properties are particularly important for graded, insulating coatings. It was noted that developing both an ability to measure thermal conductivity and to model this property based on microstructural parameters would enhance industry’s ability to design coatings for particular applications.
- Group participants suggested that round robin programs to establish a basis for comparison of test methodologies would be productive as has been shown in a recent exercise to evaluate techniques of metallographic preparation of tungsten carbide coatings. This latter effort which entailed the distribution and analysis of 1800 samples has provided evidence of the value of standard reference materials and standard evaluation techniques. In the long-term, databases on thermal, mechanical, and other properties may be feasibly developed by industry if accepted measurement and analysis techniques are available.

### 4.3 Performance Evaluation

Performance evaluation and prediction techniques are vital to the competitiveness of material producers and coating vendors and hence are usually closely held. The performance evaluation group identified several general service related issues which should guide the development of a research agenda.

- Although research on current applications is of value, attention should be directed to emerging applications with major growth potential. These applications include thermal barrier coatings for non-aircraft engine applications in the automotive industry, corrosion resistant coatings of value to the chemical industry, and electrical insulators applied to elecromechanical equipment used in various commercial products.
- Tungsten carbide/cobalt wear resistant coatings are a large thermal spray market. Less costly alternatives to this material are of interest and research to assess their performance limits would be of benefit.
- Corrosion resistant coatings for aqueous and other environments are of interest to several industries. An improved understanding of mechanisms of deterioration which would allow better material selection and performance prediction is desired. In terms of characterization techniques, the ability to measure permeability of coatings on a substrate was specifically cited.
- Measures of performance are application specific and the occurrence of unforeseen unmeasured operating conditions limit predictive capabilities. In this context, it was emphasized that understanding mechanisms of coating failure would be of value, particularly in relating laboratory assessments to field measurements.
- The ability to determine the condition and predict the remaining life of coatings is valuable. This capability and the desire to inspect coatings, without reliance on test coupons included in production lines, led to the recommendation of development of *in situ* nondestructive evaluation techniques.

In the extreme, industrial representatives stated the desire to be able to specify performance based solely on processing conditions. This is recognized as a long-term goal which requires significant understanding of the particular mechanisms of deterioration likely for an application and the role of coating properties and microstructure in that mechanism.

### 4.4 Process Modeling

Process modeling was recognized as the activity which binds several aspects of thermal spray coating together. Modeling of the process from the torch to the coating deposition was cited as necessary for process design, control, and automation. Group recommendations for research included the following specific items:

- Most modeling research has been directed to plasma spraying. The increased interest in high velocity oxygen fueled spraying argues for the development of models of this process wherein higher velocities and deposition rates present challenges.
- For all processes, models of the development or evolution of the complex microstructure are needed. Microstructural development models would provide a link between processing and properties with the potential for better property control and consistency.
- Microstructural development models should include understanding of the nature of impaction and coalescence of droplets, the formation of defects, and the fine features of bonding to the substrate.
- Modeling of thermal spray torch parameters is important for process improvement. Specifically, models of the thermal and flow behavior of the hot gases emanating from the torch and the behavior of particles in flight to the work-piece were cited as necessary.

Significantly, it was stated that the process modeling efforts should be integrated with both diagnostic developments and process design and that the specific classes of material addressed should be recommended by industry.

## 5. Conclusions

The workshop was successful in identifying many of the key problem areas in thermal spray coating technology. A broad spectrum of issues in this complex process was addressed in the discussion groups which reflected the concerns of different industries. The active participation of the attendees reflects the interest industry has in the development of a research agenda which addresses improved process reproducibility and performance prediction. It is significant to note that most attendees expressed a willingness both to identify important issues which limit the technology and to participate in collaborative research projects to which they would contribute materials, services, and expertise. A key to this willingness was the realization that industrial and academic capabilities in material processing could be effectively utilized in conjunction with NIST’s measurement, modeling, and characterization capabilities. Opportunities for transfer of research results to industry through established thermal spray technical organizations were clarified. As a result of the workshop, NIST will synthesize consensus project plans for the consideration of the attendees and initiate collaborative research where sufficient interest warrants.

## Figures and Tables

**Table 1:** Characteristics of thermal spray deposition of tungsten carbide-cobalt coatings

Typical bond strength, MPA	HVOF	Standard plasma	High velocity plasma
Flame temperature, °C	2760	11,100	11,100
Gas velocity	Mach 4	Subsonic	Mach 1
Hardness, DPH 300	1,050	750	950
Porosity, %	0	<2	<1
Typical bond strength, MP_a_	69	55	69
Thickness limit, mm	1.52	0.76	0.38
